# Development, Methodology and Potential of the New Universal Visual Scoring System (UniViSS) for Caries Detection and Diagnosis

**DOI:** 10.3390/ijerph6092500

**Published:** 2009-09-23

**Authors:** Jan Kühnisch, Inka Goddon, Susanne Berger, Helga Senkel, Katharina Bücher, Thomas Oehme, Reinhard Hickel, Roswitha Heinrich-Weltzien

**Affiliations:** 1 Department of Conservative Dentistry and Periodontology, Ludwig-Maximilians-University of Munich, Munich, 80336, Germany; E-Mails:kbuecher@dent.med.uni-muenchen.de (K.B.);hickel@dent.med.uni-muenchen.de (R.H.); 2 Health Department of the Ennepe-Ruhr-District, Schwelm, 58332, Germany; E-Mails:I.Goddon@en-kreis.de (I.G.);S.Berger@en-kreis.de (S.B.);H.Senkel@en-kreis.de (H.S.); 3 Dental practice, Lichtenstein, 09350, Germany; E-Mail:t.oehme@web.de; 4 Department of Preventive Dentistry, University of Jena, 07743, Germany; E-Mail:Roswitha.Heinrich-Weltzien@med.uni-jena.de

**Keywords:** dental caries, caries detection and diagnosis, visual examination, epidemiology

## Abstract

Given the limitations of adjunct caries detection and diagnostic tools, e.g., imperfect validity and reproducibility, as well as the difficulties in controlling all possible confounding factors, the need for an objective visual caries detection and diagnosis system has become evident. Our work has therefore aimed at systematizing caries lesions with the Universal Visual Scoring System (UniViSS) for occlusal and smooth surface lesions, which can be used for primary and permanent teeth, as well as under clinical, epidemiological, public health and laboratory conditions. Besides the description of the development and methodology of UniViSS, it is shown that UniViSS allows an accurate and reproducible classification of caries lesions on occlusal surfaces.

## 1. Introduction

Visual caries detection and diagnosis is a key area for each dental practitioners, epidemiologists, teachers and scientists. While the application of the WHO's DMF index [1] as a basic oral health measure will continue, due to its worldwide acceptance, its convenience and the possibility to compare past dental data with future findings, due to their high frequency there is a strong need to consider non-cavitated caries lesions as a relevant dental health indicator [2]. As such lesions will not be registered with the DMF index, new and more precise visual caries detection and diagnostic methods should be used to record the overall dental health status of a patient. Recently introduced diagnostic systems, e.g., the criteria by Ekstrand *et al.* [3], Nyvad *et al.* [4] and the ICDAS (International Caries Detection and Assessment System) group [5,6], have included non-cavitated caries lesions, but classify the caries process with only a few criteria [5,6]. However, due to the fact that the clinical appearance of carious lesions is complex, a limited set of criteria seems to be unlikely to describe the clinical appearance as precisely as possible. The ICDAS II criteria do no longer, for example, distinguish between white and brown discolorations [5,6], which, in fact, provide important information about lesion activity [7] and are essential for comprehensive caries monitoring in longitudinal studies. The previously used differentiation according to Ekstrand *et al.* [3,8] nevertheless proved useful and practicable in own investigations [9]. Further, the classification of lesions with both white opacities and brown discolorations on the other hand was difficult, as was distinguishing precisely between brown discolorations and microcavities. These clinical experiences, as well as the fact that adjunct diagnostic methods, e.g., laser fluorescence measurements, electrical resistance measurements and quantitative light-induced fluorescence measurements do not perform as satisfactorily on non-cavitated occlusal surfaces as was hoped [10–14] constitute the main drivers for the development of the Universal Visual Scoring System (UniViSS). Furthermore, the system should compensate for inaccuracies of existing scoring systems, fulfill the current requirements for caries detection and diagnostic methods as well as be adjustable [15]. Hence the first aim of the present work was to describe the development and methodology of UniViSS for occlusal and smooth surfaces in primary and permanent teeth. Our second aim was to analyse the validity and reproducibility of UniViSS on occlusal surfaces for the first time as both issues are of clinical importance to verify the potential of this new visual caries detection and diagnostic system on the most caries susceptible sites.

## 2. Material and Methods

### 2.1. Selection of the Diagnostic Criteria

The methodical development of UniViSS took into consideration basic diagnostic principles and well-accepted caries detection and diagnostic criteria, e.g., WHO basic methods [1], the criteria given by Ekstrand *et al.* [3], Nyvad *et al.* [4], as well as the recently introduced ICDAS criteria [5,6]. Furthermore, Ismail’s [16] systematic review on visual caries detection and diagnostic methods provided valuable information. This means that UniViSS uses clinically accepted and validated criteria such as white and brown opacities, microcavities, occurrences of enamel breakdowns and grey translucencies. But with white-brown discolorations UniViSS adds a new criterion that appears for many lesions in the clinical practice. Deep cavities with pulpal involvement were likewise considered since they are commonly associated with early childhood caries while also occurring with frequency in the permanent dentition of patients in developing countries, where caries remains often untreated. By matching them against our image database, which currently contains over 1,500 high-quality photographs of very different carious lesions from occlusal and/or smooth surfaces of primary and permanent teeth, the criteria were generalized and summarized. For 117 occlusal surfaces histological findings from a preliminary study were also available in addition to the photographs [18]. These data, together with the results of previously validated investigations [3,8,17–19], were the mainly influences for the systematization of our diagnostic system.

### 2.2. Systematization of the Diagnostic Criteria

**Figure 1.**
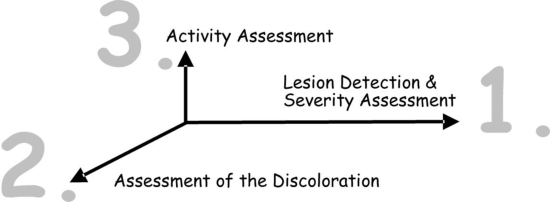
Three-step procedure of UniViSS for detection and assessment of caries lesions.

Different to existing visual systems for caries detection/diagnosis, which are essentially a sequence of criteria from ‘healthy’ to ‘severely decayed’, UniViSS uses a three-step diagnostic procedure to classify in detail the complex clinical appearance of carious lesions. These three steps are: (1) severity assessment (the severity also determines the detection level, if a caries lesion is present); (2) discoloration assessment; and (3) activity assessment. The lesion activity should be recorded as a Yes/No decision. As a whole, UniViSS can therefore be understood as a three-dimensional system of caries detection and diagnosis (Figure 1).

### 2.3. Pre-Clinical Tests on Practicability

The stepwise development of UniViSS happened over several years and was supported by repeated evaluations of the system to its present state. Each time, several experienced dentists assessed the system’s status in terms of its practicability, consistence and design under different conditions. The evaluations were followed by an exchange of knowledge and ideas and discussions about possible improvements. The development status of UniViSS was then updated and approved. The following tests were carried out:

- Examination of 40 subjects at two different points in time as part of a general screening. The examinations were done by separate examiners (I.G., S.B., H.S.).
- Evaluation of the criteria under field conditions for 12-year olds as part of the standard school examination in a provincial town of the Philippines (R.H.-W.).
- Application and assessment in a dental office (T.O.).
- On-going review of the criteria set as part of patient care in university hospitals and the training of under- and postgraduate students and dentists (J.K., R.H.-W.).

### 2.4. Validation and Reproducibility Testing of UniViSS on Occlusal Surfaces

For testing the validity of UniViSS a sample of 65 sound and mostly non-cavitated third molars was selected from a pool of teeth extracted for surgical or orthodontic reasons. Only permanent molars without sealants, fillings, cavitations, approximal and/or buccal/lingual caries lesions and developmental defects were included. All teeth were independently categorized according to the newly developed UniViSS criteria by two dentists. All diagnoses were counterchecked one week later to form a consensus diagnosis for each surface. In case of different findings, both examiners discussed the discordant results and came to an agreement. The histological validation included the sectioning of all teeth with a microtome saw (Mikrotrenn MT 1-78-03, Hofer, Switzerland) to find the maximum caries extension of the lesion. To assess the caries extension more precisely all slices were examined under a light microscope at sixteen fold magnification (Stemi SV11 stereomicroscope, Zeiss, Oberkochen, Germany). The slice with the greatest caries extension for each specimen was identified, digitally photographed and stored. Each caries lesion was classified according to the criteria by Marthaler [20]. The diagnosis for each specimen was counterchecked one week after the initial examination. In case of different findings regarding the caries extension, the examiners discussed their discordant measures to reach an agreement. Both examiners were blinded to the visual appearance of each surface. The validity of UniViSS was determined by calculating the sensitivity (SE), specificity (SP) and the Az values under the ROC curves for the caries detection level (D0 vs. D1-4) as well as for the dentine caries detection level (D0-2 vs. D3-4).

For testing intra- and inter-examiner reproducibility the inaugurator of UniViSS (J.K.) as well as two other dentists (D1, D2) investigated 60 sound and non-cavitated extracted third molars under standardized *in vitro* conditions. Prior to the beginning of all measurements all examiners were introduced to the use of UniViSS and received extensive theoretical and practical training. Each evaluation cycle was repeated after a minimum interval of two weeks to safeguard the blindness of each investigator between both measurement cycles. Weighted Kappa values (wK) and the percentage of coincident readings were calculated as measure of agreement for categorical data to determine the intra- and inter-examiner reproducibility. The reproducibility can be assessed as low for wK below 0.40, moderate for wK between 0.41 and 0.60, good for wK between 0.61 and 0.80 and excellent for wK between 0.81 and 1.00.

## 3. Results

**Figure 2.**
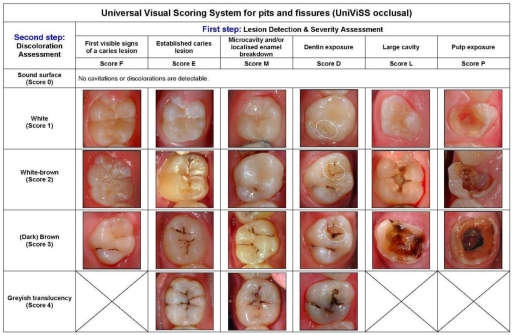
Criteria of the Universal Visual Scoring System for pits and fissures (UniViSS occlusal).

**Figure 3.**
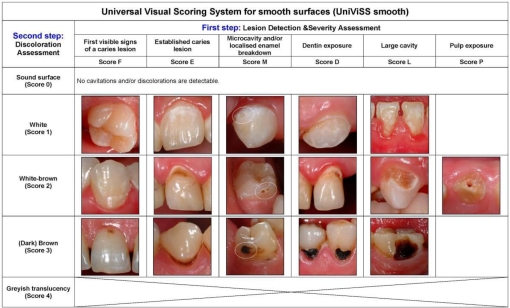
Criteria of the Universal Visual Scoring System for smooth surfaces (UniViSS smooth).

Our efforts led finally to the development of a visual system for the detection and diagnosis of caries that is universally applicable and adaptable (Figure 2 and 3, detailed descriptions of each criteria can be taken from the electronic supplementary information). The clinical use of the system is very flexible and may be adjusted as required. It is not only possible to choose the most appropriate criteria set for each study protocol but the examination conditions can also be modified according to what is intended to be achieved. Table 1 summarizes the typical parameters. For a better comparability of results the selected examination details should be specified in each study protocol. The standard examination equipment consists of the CPI probe and the dental mirror. The CPI probe is used to help the metric differentiation between established lesions and microcavities as well as for the pressureless tactile examination of the tooth surface.

For analyzing the caries activity lesion-related factors, e.g., discoloration, location on the tooth surface, presence or absence of plaque and superficial texture/roughness play an important role in addition to patient-related factors such as age, individual caries risk, oral hygiene, tooth-friendly nutrition and fluoride intake. White and white-brown colored lesions, plaque coverage, roughness and the location in plaque retentive areas are strong indicators of a lesion which is active (Table 2).

According to the second aim of this study, the validity and reproducibility of UniViSS on occlusal surfaces was tested. Histological validation revealed that 12 teeth (18%) showed no signs of demineralization, 30 teeth (46%) had a caries in the enamel (D1-2), 17 teeth (26%) showed a lesion limited to the outer half of the dentin (D3), whereas in six teeth (10%) caries had already spread into the inner half of the dentin (D4). After correlating the histological findings with UniViSS the following data were obtained for the caries detection level (D0 vs. D1-4): SE 100.0%, SP 58.3% and Az 0.841. For dentine caries detection (D0-2 vs. D3-4) the parameters were SE 62.5%, SP 97.6 and Az 0.816. The intra-examiner wK values/coincident readings were found within a range between 0.90/93% (D1, D2) and 0.98/98% (J.K.); the inter-examiner reproducibility amounted to 0.89/91% (D2), 0.97/98% (D1) and 0.93/95% (J.K.).

## 4. Conclusions

This manuscript describes the development and methodology of UniViSS. Firstly validity and reproducibility data for occlusal surfaces is presented. The development of UniViSS was mainly influenced by the results of previous epidemiological and diagnostic caries studies by the authors [2,9,10,12–14,21–23], the huge data base of high-quality photographs from caries lesions, the clinical experience of the authors as well as the empirical data obtained during the tests on practicability. Using this methodology, the inaccuracies of existing visual diagnostic systems mentioned in the introductory section were compensated. Another advantage worth stressing is the consistency of the criteria for occlusal and smooth surfaces, as well as for primary and permanent teeth. For smooth surfaces the system’s availability extends to the freely accessible vestibular and oral as well as to the approximal surfaces. It also enables the visual caries detection and diagnosis of caries-related cervical defects in older patients. This means that the system can non-restrictively be used in all age groups.

After analyzing the diagnostic performance it can be concluded that the validity and reproducibility results are very encouraging. Considering the registered validity data in comparison to previously published results of different visual caries detection and diagnostic systems [4–6,8,24], the documented Az values for the overall caries detection level (0.84) and for the dentine caries detection level (0.82) are in the same range. According to our results the intra- and inter-examiner-reproducibility can be assessed as excellent after an extensive theoretical and practical training. The registered wK values were found to be in the same order of magnitude that was obtained for the visual criteria by Ekstrand *et al.* [3,8] or the ICDAS [5,6].

When reflecting of the methodology and potential of UniViSS for dental practice, clinical studies and epidemiological trials the new visual caries detection and diagnostic system allow a precise description of non-cavitated and cavitated caries lesions alike. To make examination data comparable UniViSS follows basic principles [1] and is considering most frequently used and validated criteria [3,5,6,8,20], too. In this context, UniViSS should be understood as an addition to existing methods, used to visually describe the clinical appearance of (non-)cavitated carious lesions as precise as possible. According to the authors, it is only possible under this assumption that (non-)cavitated lesions can be detected and diagnosed reliably, that the diagnostic performance of visual methods can be improved, that changes of carious lesions can be detected as part of a caries monitoring and that caries activity can be assessed most accurately. In order to assess the activity of carious lesions with UniViSS the degree of discoloration as well as the clinical conditions from Table 2 need to be taken into account. As a general assumption, white and white-brown lesions are associated with more active caries lesions, while brown and black lesions normally indicate a slower disease progression. Apart from discolorations, plaque is another etiological factor that also provides clues to the activity of a caries lesion. Location plays a part here, too. The natural plaque stagnation areas [17], e.g., the vestibular surfaces nearby the gingival margin, the lingual surfaces of the molars in the lower jaw, or the approximal spaces, are difficult to reach with the tooth brush and therefore more likely to be caries susceptible. Whether roughness will gain significance as a valid caries predictor in the future remains to be seen [17].

Due to the meticulous classification of all possible stages of the caries process UniViSS is able to register logically the clinical appearance of all kind of carious lesions. However, by grouping the various criteria its use can be simplified significantly, e.g., for the purpose of epidemiologists. Epidemiological studies normally require a simple and practicable procedure to ensure that the several tests can be carried out efficiently under field conditions, too. The easiest way of doing this is to group all non-cavitated lesions under the same category (Scores F, E, M) apart from registering the cavitated caries lesions (Scores D, L, P = D-component of the DMF index) and to record them as one score for the respective tooth or surface. Thus, any carious processes that can at least be observed visually will be captured. Although it requires more effort, each of the three diagnostic steps mentioned above (1. severity assessment, 2. discoloration assessment, and 3. activity assessment) may also be included in the study design. In order to ensure the reproducibility of the results the teeth should be cleaned and the examiner(s) should have received a calibration training prior to the study.

Each caries diagnosis has to be linked with distinct treatment strategy. Therefore the dentist has to decide whether those lesions may be treated with non-invasive/preventive or invasive/restorative measures. The classification according to the treatment strategies suggested by Pitts [25]—non-active care advised, preventive care advised and operative care advised—is therefore sufficient. Since every diagnosis should be linked to specific treatment strategies, the clinical consequences of different UniViSS results will be outlined in the following. While active treatment is not considered necessary as such for sound tooth surfaces, the intensification of preventive efforts at home remains a basic aim for caries risk patients as well as on existing caries lesions. First visible lesions as well as established lesions should be treated with professional measures, i.e., topical fluoridation and the sealing of pits and fissures, in addition to a more comprehensive home prophylaxis program, to stop caries progression. On microcavities—if the radiograph shows an involvement of the dentine which indicate a bacterial infection—an appropriate restorative treatment seems to be indicated in most of the cases [26]. Otherwise, the complete sealing of the microcavity constitutes a possible therapeutic option [27–29]. Restoration is clearly indicated for carious lesions with dentine exposure, greyish translucencies or gross cavitations. The decision between endodontic therapy or extraction after pulp exposure should be carefully weighted based on patients preferences, the individual dental health status, age as well as the need, feasibility and costs for follow-up treatments. The measures listed here represent mainly the preventive and therapeutic spectrum for industrialized countries, whereas fundamentally different strategies might be applicable for similar diagnostic findings in developing countries with limited resources.

The present development state of UniViSS allow a precise recording of the entire spectrum of (non-)cavitated caries lesions on occlusal and smooth surfaces with just one visual diagnostic system. After the systematization of the criteria and the first encouraging assessment of the diagnostic performance further studies can be planned.

## Figures and Tables

**Table 1.** Summary of possible conditions for the usage of UniViSS.

**Study type**	**Tools**	**Illumination**	**Tooth cleaning**
Field	Cotton rolls	Head lamp	Patient brushed him/herself
Clinical	Magnifying glass/loop	Examination lamp	Professional tooth cleaning with a bristle brush etc.
Laboratory	Dental unit with air syringe	Operating light of the dental unit	Professional tooth cleaning with air polishing

**Table 2.** Clinical indicators of inactive and active caries lesions on occlusal and smooth surfaces.

**Occlusal pits and fissures**		**Smooth surfaces**	
**Inactive**	**Active**	**Inactive**	**Active**
Persistence of the lesions over years/decades	Detection within some years after tooth eruption	Persistence of the lesions over years/decades	Detection within some years after tooth eruption
No plaque coverage	Plaque coverage	No plaque coverage	Plaque coverage
Glossy, shiny appearance of the enamel surface after air-drying	Matt/frosty/rough appearance of the enamel surface after air-drying	Glossy, shiny appearance of the enamel surface after air-drying	Matt/frosty/rough appearance of the enamel surface after air-drying
No pathological enlargements	Microcavities	Lesions located in distance to the gingiva	White spots near the gingiva margin
Brown discoloration in enamel	White(-brown) discoloration in enamel	Brown discoloration in enamel	White(-brown) discoloration in enamel
Hard, dry, discolored dentine	Soft, wet, (un)discolored dentine	Hard, dry, discolored dentine	Soft, wet, (un)discolored dentine
